# Impact of age on early outcome after coronary bypass graft surgery using minimized versus conventional extracorporeal circulation

**DOI:** 10.1186/s13019-014-0143-3

**Published:** 2014-08-28

**Authors:** Philipp Kolat, Michael Ried, Assad Haneya, Alois Philipp, Reinhard Kobuch, Stephan Hirt, Michael Hilker, Christof Schmid, Claudius Diez

**Affiliations:** Department of Cardiothoracic Surgery, University Medical Center Regensburg, Franz-Josef-Strauss Allee 11, Regensburg, 93053 Germany; Department of Cardiac and Vascular Surgery, University Medical Center Schleswig Holstein, Arnold-Heller-Straße 3, Kiel, 24105 Germany

**Keywords:** Coronary artery bypass grafting, Minimized extracorporeal circulation, Mortality, Outcome

## Abstract

**Background:**

Objective of this study was to evaluate the impact of age on comparative early outcomes after coronary artery bypass graft surgery (CABG) with minimized (MECC) and conventional extracorporeal circulation (CECC).

**Methods:**

A retrospective age-, gender- and operation-matched cohort analysis between January 2005 and December 2010 with a total of 2274 patients undergoing CABG with MECC (n = 1137; 50%) or CECC was performed. Patients were stratified into 4 groups according to age: <59 years, 60–69 years, 70–79 years, and 80 years of age or older. Outcomes were compared within each age group. Patients with preoperative dialysis were excluded from analysis. Primary endpoint was 30-day mortality.

**Results:**

Patients treated with CECC had a significantly higher mean logistic EuroSCORE (6.3% vs. 5.0%; p < 0.001), a slightly lower rate of preoperative myocardial infarction (46% vs. 51%; p = 0.01) and a higher rate of impaired renal function (eGFR < 60 mL/min/1.73 m2: 24% vs. 20%; p = 0.01) compared to MECC-patients. Left internal mammary artery was significantly used more often in MECC patients (93% vs. 86%; p < 0.001). Cardiopulmonary bypass and aortic-cross clamping time were significantly lower in the MECC group (p < 0.001). Overall 30-day mortality was significantly higher in patients treated with CECC (4.4% vs. 2.2%; p = 0.002). Within the different age groups mortality rates were not significantly different except for patients aged 60–69 years (4.5% vs. 1.8%; p = 0.03). Postoperative requirement of renal replacement therapy (4% vs. 2.2%; p = 0.01), respiratory insufficiency (9.9% vs. 6.6%; P = 0.004) and incidence of low cardiac output syndrome (3% vs. 1.2%; p = 0.003) were significantly increased in patients with CECC. Multivariate analysis identified age (p = 0.005; 95% CI 1.01 to 1.08; OR 1.05) among other parameters as an independent risk factor, whereas conventional extracorporeal circulation itself did not present as an independent risk factor for 30-day mortality.

**Conclusions:**

In this matched study sample early outcome was significantly better in patients with MECC compared to CECC, irrespective of age. Prior myocardial infarction estimated GFR < 60 mL and waiving the use of LIMA were independent risk factors for 30-day mortality, which were more present in the CECC group.

## Background

Conventional extracorporeal circulation (CECC) is still the gold standard perfusion technique for on-pump coronary artery bypass grafting (CABG). It is world-widely established with standardized proceedings and therefore safe with low related mortality [1]-[3]. Nevertheless, it is also associated with serious complications such as stroke, hemodilution, coagulopathy, renal dysfunction, and systemic inflammatory response syndrome (SIRS) [4],[5].

Beating-heart or off-pump CABG has gained attention as an alternative to myocardial revascularization with CECC over the recent years. Unfortunately, the procedure is technically demanding for cardiac surgeons and preferably used, if not all three coronary vessels must be provided with bypasses [6]-[8].

Initially, the minimal extracorporeal circulation system (MECC) was developed to facilitate beating heart revascularization, i.e. to maintain the benefits of beating heart surgery and minimize the disadvantages of on-pump revascularization. The MECC system is a closed, fully heparin-coated and pre-connected ECC system, basically consisting of a diffusion membrane oxygenator and a centrifugal pump. The crucial advantage is a higher biocompatibility. It has been proposed that MECC might reduce the harmful effects of CECC, such as systemic inflammatory reaction, hemolysis, hemodilution, disturbances of blood clotting disorders and other postoperative complications. Several authors analyzed the impact of MECC in special patient-cohorts with diabetes, renal insufficiency or in emergent cases [9]-[11].

The aim of this study was to evaluate the impact of age on comparative early outcomes after coronary artery bypass graft surgery with minimized and conventional extracorporeal circulation.

## Methods

This study was based on a retrospective analysis of 2274 consecutive patients undergoing CABG with MECC (n = 1137; 50%) or CECC between January 2005 and December 2010 at the Department of Cardiothoracic Surgery, University Medical Center Regensburg, Germany. Whether CECC or MECC was used, was up to the surgeons’ preferences and the availability of the perfusion systems.

The study was performed as an age-, gender- and operation-matched cohort analysis. Patients were divided into 4 groups according to age: group 1 < 59 years, group 2 60–69 years, group 3 70–79 years, and group 4 80 years of age or older. Postoperative outcome was compared within each age group. Patients with preoperative dialysis were excluded from analysis. Primary endpoint of this study was 30-day mortality.

The extracorporeal circuit of the standard ECC consisted of an open system with a non-heparin-coated tube system. A 39-50 F two-stage cannula (Stöckert, Germany) was used to drain the venous blood from the right atrium, a 22 F aortic cannula (Maquet, Germany) for the distal ascending aorta. The MECC was a closed system with a significantly reduced priming volume without contact of blood with air. The components of the system included a membrane oxygenator (Quadrox D; Maquet, Germany), a centrifugal pump, a table line (3/8 inch, 180 cm), a venous two-stage cannula (32–40 F) and an aortic cannula (21 F). For further detailed information concerning both systems, we refer to previous publications [12]-[15].

All operations were performed by senior cardiac surgeons, who were experienced with both, MECC and CECC. The proportion of MECC procedures did not significantly differ between surgeons and all also operated a similar proportion of patients with CECC. The final decision whether to use MECC or not was left to the surgeon.

The statistical analysis was performed using the SPSS 11.0 software (SPSS Inc., Chicago, IL). Data are presented as mean values + their first standard deviation, or, where appropriate and indicated, as median and interquartile range (IQR). Categorical variables are displayed as frequency distributions (n) and simple percentages (%). Multivariate analysis of statistically significant parameters was used for detection of independent risk-factors for 30-day mortality. A p-value of less than 0.05 was considered significant.

## Results

### Demographic data

Detailed demographic data including co-morbidities can be obtained from Table 1. Patients treated with CECC had a significantly higher mean logistic EuroSCORE (6.3% vs. 5.0%; p < 0.001), with significant lower ejection fraction (60% vs. 63%; p < 0.001). The MECC group showed a lower rate of atrial fibrillation (75% vs. 44%; p = 0.002), but a slightly higher rate of preoperative myocardial infarction (46% vs. 51%; p = 0.01). Renal function was more impaired in the CECC-group (estimated GFR < 60 mL/min/1.73 m^2^: 24% vs. 20%; p = 0.01) compared to MECC-patients. Priority for cardiac surgery was homogeneously distributed between the two groups.

**Table 1:** **Demographic data**

Variable	CECC	MECC	p-value
	(n = 1,137)	(n = 1,137)	
Male gender [n; %]	995 (80.4)	995 (80.4)	1.00
Age [years]	67.3 ± 8.8	67.3 ± 8.8	1.00
Age group [n; %]			
< 59 (group 1)	240 (19)	240 (19)	1.00
60 – 69 (group 2)	440 (36)	440 (36)	
70 – 79 (group 3)	485 (39)	485 (39)	
> 80 (group 4)	72 (6)	72 (6)	
Logistic EuroSCORE [%, 95% CI)	6.3 (5.8 to 6.6)	5.0 (4.6 to 5.3)	< 0.001
Ejection fraction [%]	60 (47; 70)	63 (50; 70)	< 0.001
Height [cm]	171 ± 8.5	171 ± 8	0.26
Weight [kg]	86 ± 16	82 ± 13	< 0.001
Atrial fibrillation [n; %]	75 (6.1)	44 (3.3)	0.002
COPD [n; %]	107 (8.6)	94 (7.4)	0.24
Active smoker [n; %]	387 (31)	355 (29)	0.35
Preoperative myocardial infarction [n; %]	564 (46)	627 (51)	0.01
Insulin-dependent diabetes mellitus [n; %]	148 (12)	138 (11)	0.57
Diabetic nephropathy [n; %]	52 (4.2)	47 (3.8)	0.68
Serum creatinine [mg × dL^−1^]	1 (0.8; 1.2)	0.9 (0.8; 1.1)	0.10
Estimated GFR [mL × min^−1^ × 1,73 m^−2^]	79 (62; 96)	82 (64; 100)	0.02
Estimated GFR < 60 mL × min^−1^ × 1,73 m^−2^ [n; %]	297 (24)	243 (20)	0.01
Indication for surgery [n; %]			< 0.001
Elective	674 (55)	588 (48)	
Urgent	368 (30)	496 (40)	
Emergency	195 (16)	153 (12)	
Second cardiac operation [n; %]	91 (7.4)	14 (1.1)	< 0.001

### Perioperative data

The number of grafts was significantly higher in patients with MECC (p = 0.004). Left internal mammary artery was also used more often in MECC patients (93% vs. 86%, p < 0.001). Cardiopulmonary bypass- and aortic-cross clamping time were significantly lower in the MECC group (p < 0.001). Interestingly, there was no difference concerning drainage loss between the two groups (Table 2).

**Table 2:** **Peri- and postoperative data**

Variable	CECC (n = 1,137) [group A]	MECC (n = 1,137) [group B]	p-value
No of grafts [n]	3 (2; 3) Range (1 – 6)	3 (2; 4) Range (1 – 6)	0.004
Use of LIMA [n; %]	1061 (86)	1150 (93)	< 0.001
Bypass time [min]	93 (75; 116)	78 (60; 97)	< 0.001
Aortic cross clamp time [min]	53 (44; 66)	47 (35; 60)	< 0.001
Drainage-loss [mL]	550 (350; 800)	550 (370; 850)	0.33
Resternotomy [n; %]	67 (5.4)	69 (5.6)	0.93
Mechanical ventilation [hours]	12 (9; 16)	11 (8; 15)	0.34
Serum creatinine postoperative [mL × min^−1^ × 1,73 m^−2^]	1 (0.9; 1.3)	0.9 (0.8; 1.2)	< 0.001
Serum creatinine at discharge [mL × min^−1^ × 1,73 m^−2^]	1.1 (0.9; 1.4)	1 (0.9; 1.3)	< 0.001
Postoperative dialysis [n; %]	49 (4.0)	27 (2.2)	0.01
< 59 (group 1)	6 (2.5)	2 (0.8)	0.29
60 – 69 (group 2)	17 (3.9)	8 (1.8)	0.10
70 – 79 (group 3)	20 (4.1)	12 (2.5)	0.21
> 80 (group 4)	6 (8.3)	5 (6.9)	1.0
Low cardiac output syndrome [n; %]	37 (3)	15 (1.2)	0.003
Central neurologic event [n; %]	26 (2.1)	16 (1.3)	0.16
Respiratory insufficiency [n; %]	122 (9.9)	82 (6.6)	0.004
Reintubation [n; %]	36 (2.9)	30 (2.4)	0.53
Pneumonia [n; %]	64 (5.2)	42 (3.4)	0.04
ICU-stay [days]	1 (1; 3)	1 (1; 2)	< 0.001
Hospitalization [days]	11 (9; 14)	11 (9; 13)	0.22
In-hospital mortality [n; %]	51 (4.1)	21 (1.7)	< 0.001
30-day mortality [n; %]	56 (4.5)	27 (2.2)	0.002
≤ 59 (group 1)	8 (3.3)	2 (0.8)	0.11
60 – 69 (group 2)	22 (4.5)	8 (1.8)	0.03
70 – 79 (group 3)	20 (4.1)	13 (2.6)	0.29
≥ 80 (group 4)	8 (11.1)	4 (5.6)	0.37

### Postoperative mortality and outcome

Detailed information can be obtained from Table 2. Duration of postoperative mechanical ventilation showed no significant difference between CECC and MECC (although respiratory insufficiency and pneumonia was more common in CECC patients), as well as need for re-sternotomy. Postoperative as well as discharge serum creatinine were significantly lower in the MECC group (p < 0.001), therefore, need for dialysis was necessary in CECC group more often (p = 0.01), but was not dependant on age.

Low cardiac output syndrome with prolonged inotropic support was more common in CECC patients (p = 0.003). ICU-stay was significantly longer in the CECC group (p < 0.001), but did not have an influence on total hospital-stay (p = 0.22). Overall 30-day mortality was significantly higher in patients treated with CECC (4.4% vs. 2.2%, p = 0.002). Within the groups 1 to 4, mortality rates were not significantly different except for patients aged 60–69 years (4.5% vs. 1.8%; p = 0.03).

Multivariate analysis identified prior myocardial infarction infarction (p = 0.001; 95% CI 1.47 to 4.33; OR 2.53), estimated GFR < 60 mL (p = 0.001; 95% CI 1.41 to 3.86; OR 2.33) and waiving the use of LIMA (p = 0.001; 95% CI 1.49 to 4.73; OR 2.65) to be independent risk factors for 30-day mortality, whereas CECC and reoperation itself was not associated with a higher rate (Table 3).

**Table 3:** **Multivariate analysis: independent risk-factors for 30-day mortality**

Variable	p-value	Odds ratio	95%CI
Preoperative atrial fibrillation	0.49	0.64	0.18 – 2.28
Preoperative myocardial infarction	0.001	2.53	1.47 – 4.33
Estimated GFR < 60 mL × min^−1^ × 1,73 m^−2^]	0.001	2.33	1.41 – 3.86
Second cardiac operation	0.94	0.97	0.42 – 2.24
CECC	0.189	1.43	0.84 – 2.45
No LIMA use	0.001	2.65	1.49 – 4.73
Bypass time [min]	0.001	1.02	1.02 – 1.03
Aortic cross clamp time [min]	0.001	0.97	0.96 – 0.98

## Discussion

Demographic changes over the last decades led to a different patient population, not only for cardiac surgeons. Todays’ elderly patients are characterized by multiple, partially severe co-morbidities including hypertension, diabetes, pulmonary diseases, renal insufficiency, obesity as well as peripheral arterial disease (PAD). The informed patient’s wish to benefit from high-end medicine is not only a social-economic burden, but also a demanding challenge for cardiac surgeons. The trend is expected to continue [16].

Conventional extracorporeal circulation (CECC) is still the preferred perfusion technique for on-pump coronary artery bypass grafting (CABG). It is world-widely established with standardized proceedings and therefore safe with low related mortality [1]-[3]. The minimal extracorporeal circulation system (MECC) was developed as an aid to beating heart revascularization, i.e., to maintain the benefits of beating heart surgery and minimize the disadvantages of on-pump revascularization. The use of MECC/CECC is controversely discussed, several comparisons between these two systems can be found in the recent literature, including emergency cases, diabetic patients and those with impaired renal function [9]-[11]. From our knowledge there is currently no analysis of the impact of age. Repeat revascularization occurred more frequently after off-pump CABG than after on-pump CABG [17] and off-pump CABG was associated with a higher rate of incomplete revascularization and an inferior outcome at 1 year [18].

Our trial does not support the assumption that off-pump CABG can improve the early outcome in high-risk patients [17].

The EuroSCORE [19], an established method of calculating predicted operative mortality for patients undergoing CABG, was significantly higher in the CECC group accompanied by lower ejection fraction and lower estimated GFR. For safety reasons (unskilled medical assistant, anaesthesiologist or perfusionist), as well as to manage expected high blood loss due to antiplatelet-agents in large doses, some surgeons decided to use the conventional method, but it was always up to their own preferences. Understandably, that CECC was also used more often for re-operation cases. Interestingly, we found a higher prior myocardial infarction rate in the MECC group. This reflects the fact that it was no matter of preoperative conditions whether CECC or MECC was used. Our data also suggest no significant difference in survival and outcomes in emergency patients who underwent CABG with MECC/CECC. These data are also proven by our group with respect to emergency patients, who underwent CABG with MECC/CECC; no significant difference concerning survival and outcome could be observed [9].

We could present that the MECC group had a greater number of grafts performed. In contrast, Girerd et al. [20] reported that complete revascularization does not seem to improve long-term survival in older patients and suggested that those patients at high operative risk may be considered, when deemed clinically appropriate, for limited coronary revascularization. However, we always aimed at a complete revascularization, including the use of a LIMA. Nevertheless, clamping time as well as bypass time was lower than in the CECC group, due to lacking necessity of a regular reperfusion period.

Acute kidney injury (AKI) is a main complication after on-pump coronary artery bypass graft surgery [13], which is also seen in this study. Postoperative renal function was more impaired in the CECC group with higher serum creatinine levels as well as need for more frequent dialysis. Benedetto et al. could also show that mini-CPB is associated with a lower incidence of AKI (mainly caused by better organ- and tissue perfusion), when compared with conventional CPB among patients undergoing CABG [13]. This was also observed by Skrabal et al. [21]. Our study group could already prove that MECC is renoprotective in the early postoperative period, but unfortunately cannot prevent AKI [10].

LCOS with need for inotropic support was more common in the CECC group. This can be explained by a higher EuroSCORE and a lower ejection fraction. Therefore, it is not surprisingly that CECC patients had a longer ICU stay than the corresponding matched group.

Interestingly, we could not detect a significant difference concerning the drainage loss. Many studies proposed higher blood loss and the more frequent use of red blood cell concentrates (RBC) when using the standard ECC. This could not be proven in this cohort, possibly due to strict postoperative coagulation regimens on ICU with recurrent activated-clotting-time (ACT)-controls and, if necessary, standardized counteractions. Our study group proved in former publications a significantly lower need for transfusion of RBC and FFP as well as improved blood cell preservation [9],[11],[13],[14].

Multivariate analysis revealed several independent risk factors for 30-day mortality, among them prior myocardial infarction as well as an estimated GFR < 60 mL. Besides, we could also detect bypass-/aortic cross clamp time as well as waiver of LIMA-use as independent IRFs. The use of conventional ECC did not play a role concerning this topic. We conclude that MECC is a safe procedure, although the mechanism why it is associated with lower mortality is still speculative [9].

### Limitations

This study suffers from two main limitations: first, this study is based on a retrospective and non-randomized design. All patients from our institutional database were screened to select and match patients by age, gender and kind of operation. Thus, there may be a risk of selection bias. Furthermore, as patient groups were matched by their chronological age - and not by the severity of illness and the number of co-morbidities, respectively, the impact of age per se cannot be investigated. Thus, pairs could have been also matched according to frailty (“clinical age”) of patients - and then, in a second step, the impact of (“chronological”) age on early outcomes may be assessed. Second, we caused a bias by the surgeons’ own preferences. There is no standard protocol given, if a patient is surely operated with the help of a MECC- or a CECC-system. Despite its limitations, our study provides valuable data and insight: we could demonstrate that isolated CABG surgery can be safely performed in elderly patients despite of multiple co-morbidities.

## Conclusions

In this matched study sample early outcome was significantly better in patients with MECC compared to CECC, irrespective of age. Prior myocardial infarction, waiver of LIMA-use as well as an estimated GFR < 60 mL were independent risk factors for 30-day mortality, but not the use of CECC.

## Consent

Written informed consent was obtained from the patients for the publication of this report and any accompanying images.

## Abbreviations

ACT: Activated clotting time
AKI: Acute kidney injury
CABG: Coronary artery bypass grafting
CECC: Conventional extracorporeal circulation
CI: Confidence interval
COPD: Chronic obstructive pulmonary disease
CPB: Cardio-pulmonary bypass
ECC: Extracorporal circulation
eGFR: Estimated glomerular filtration rate
FFP: Fresh frozen plasma
ICU: Intensive care unit
IQR: Interquartile range
IRF: Independent risk factor
LCOS: Low cardiac output syndrome
LIMA: Left internal mammary artery
MECC: Minimized extracorporeal circulation
OR: Odds ratio
PAD: Peripheral artery disease
RBC: Red blood cells
SIRS: Systemic inflammatory response syndrome
